# Bleomycin hydrolase regulates the release of chemokines important for inflammation and wound healing by keratinocytes

**DOI:** 10.1038/s41598-019-56667-6

**Published:** 2019-12-31

**Authors:** Rebecca Riise, Lina Odqvist, Johan Mattsson, Susan Monkley, Suado M. Abdillahi, Christian Tyrchan, Daniel Muthas, Linda Fahlén Yrlid

**Affiliations:** 1 Bioscience COPD/IPF, Research and Early Development, Respiratory, Inflammation and Autoimmune (RIA), BioPharmaceuticals R&D, AstraZeneca, Gothenburg, Sweden; 2 Translational Science & Experimental Medicine, Research and Early Development, Respiratory, Inflammation and Autoimmune (RIA), BioPharmaceuticals R&D, AstraZeneca, Gothenburg, Sweden; 3 Medicinal Chemistry, Research and Early Development, Respiratory, Inflammation and Autoimmune (RIA), BioPharmaceuticals R&D, AstraZeneca, Gothenburg, Sweden

**Keywords:** Chronic inflammation, Molecular biology

## Abstract

Bleomycin hydrolase (BLMH) is a well-conserved cysteine protease widely expressed in several mammalian tissues. In skin, which contains high levels of BLMH, this protease is involved in the degradation of citrullinated filaggrin monomers into free amino acids important for skin hydration. Interestingly, the expression and activity of BLMH is reduced in patients with atopic dermatitis (AD) and psoriasis, and BLMH knockout mice acquire tail dermatitis. Apart from its already known function, we have discovered a novel role of BLMH in the regulation of inflammatory chemokines and wound healing. We show that lowered BLMH levels in keratinocytes result in increased release of the pro-inflammatory chemokines CXCL8 and GROα, which are upregulated in skin from AD patients compared to healthy individuals. Conditioned media from keratinocytes expressing low levels of BLMH increased chemotaxis by neutrophils and caused a delayed wound healing in the presence of low-level TNFα. This defective wound healing was improved by blocking the shared receptor of CXCL8 and GROα, namely CXCR2, using a specific receptor antagonist. Collectively, our results present a novel function of BLMH in regulating the secretion of chemokines involved in inflammation and wound healing in human keratinocytes.

## Introduction

The outermost layer of human skin, the epidermis, consists of only a few layers of cells in depth and functions as a primary barrier covering of the body^1^. Structurally, the epidermis is organized into layers of keratinocytes, which through a stepwise maturation process migrates toward the surface and convert into corneocytes composing the stratum corneum^2,3^. During their outward transit, keratinocytes produce the lipid and protein components necessary for establishing an intact epidermal barrier^4^.

Proteases are important for modulating many physiological activities in the epidermis, with desquamation of the stratum corneum corneocytes being a major process to maintain the barrier function^5^. During this late stage of keratinocyte differentiation, the large, insoluble structural protein pro-filaggrin is fully degraded into free amino acids in a multistage process carried out by the proteases caspase-14, calpain I, elastase 2 and BLMH^2,6^. This generates natural moisturizing factors, trans-urocanic acid and pyrrolidone carboxylic acid, essential for epidermal hydration, acidification and protection against UV radiation^2^. BLMH is responsible for the very last step of the pro-filaggrin degradation process, namely the cleavage of citrullinated filaggrin monomers and BLMH knockout mice show impaired filaggrin processing, with defective texture and stiffness of corneocytes^7^. This cytoplasmic protease was first discovered due to its capacity to inactivate the antitumor drug bleomycin, hence its name. Although bleomycin is effective as an anti-cancer agent its therapeutic use is limited due to pulmonary toxicity. It has been shown in patients with Hodgkin’s lymphoma^8^ and testicular germ cell cancer^9^ that genetic variations of BLMH can influence disease outcome upon bleomycin treatment. While known to be expressed in most human tissue types, the physiological role of BLMH is not well understood. Studies have reported on its involvement in antigen-processing for MHC class I molecules^10^, protection against homocysteine thiolactone toxicity^11,12^ and regulation of proteins involved in neurodegeneration, such as amyloid precursor protein^13,14^. Interestingly, the generation of mice lacking BLMH demonstrated an unusual dermopathology, namely tail dermatitis with abundant neutrophil infiltration in the epidermis^15^. Furthermore, recent studies show a decreased BLMH expression and activity in skin lesions obtained from patients suffering from atopic dermatitis (AD)^16,17^ as well as psoriasis^18^.

In a meta-analysis of 7 different AD cohorts, we see inverse correlation in gene expression between BLMH and two inflammatory chemokines CXCL8 and GROα in lesions from patients compared to healthy controls. This prompted us to investigate the potential link between BLMH and the inflammatory phenotype described in AD patients. Knock-down of BLMH in a keratinocytic cell line resulted in an increased release of neutrophilic chemoattractants CXCL8 and GROα, with ensuing chemotaxis by neutrophils *in vitro*. A lowered function of BLMH also resulted in impaired wound healing by keratinocytes in an inflammatory environment, mediated by CXCR2 receptor signalling. Together, these findings indicate a novel role for BLMH in controlling the secretion of pro-inflammatory chemokines and contribution to maintenance of an intact epidermal integrity in human skin.

## Results

### BLMH gene expression is suppressed in atopic dermatitis and correlates with increased CXCL8 and GROα in patient skin lesions

**Figure 1:**
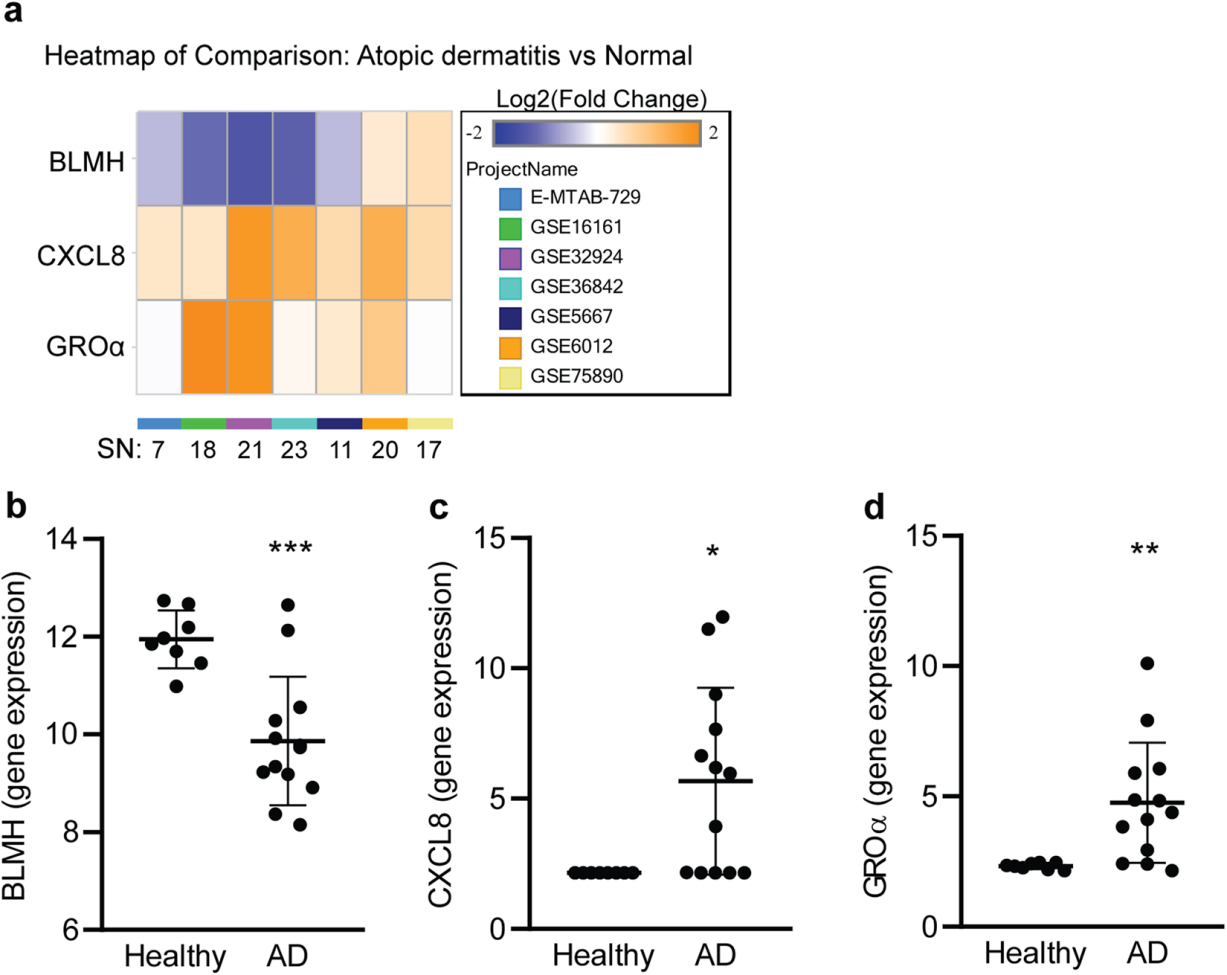
Expression of BLMH is downregulated in skin lesions from AD patients. (**a**) A genomic meta-analysis of gene expression of BLMH, CXCL8 and GROα in lesions from AD patients versus healthy individuals were conducted covering 7 cohorts with stated sample numbers (SN) of healthy individuals + AD patient samples. The Project named GSE32924 contained the largest number of AD patients and the gene expressions in patients versus healthy skin were compared in graphs (**b**–**d**) (healthy n = 8, AD n = 13, unpaired two-tailed Student’s t-tests). All values represent individual experiments with mean ± standard deviation. Significant P-values are presented as *p < 0.05; **p < 0.01; ***p < 0.001 and ****p < 0.0001.

To study how BLMH expression is affected by the chronic inflammatory environment in AD, we used the DiseaseLand transcriptomic database to perform a meta-analysis of 7 AD cohorts (Table 1) and discovered a reduction of BLMH gene expression correlated with increased CXCL8 and GROα in skin from AD patients compared to healthy individuals in the majority of the clinical studies (Fig. 1A). The cohort enrolling most AD patients (GSE32924) was further evaluated and showed a significant reduction of BLMH with induced levels of CXCL8 and GROα in patients compared to healthy lesional biopsies (Fig. 1B–D).

**Table 1:** Subject characteristics.

Project name	Disease state	Number of subjects		BLMH	CXCL8	GROα	Tissue sample	Reference
E-MTAB-729	Healthy	4	Log2 Fold change	−0.54	0.44	−0.04	Skin	Rebane *et al*.^37^
	AD	3	p-value	3.00E-02	7.04E-01	7.13E-01	Skin	
GSE16161	Healthy	9	Log2 Fold change	−1.17	0.84	2.20	Skin	Guttman-Yassky *et al*.^38^
	AD	9	p-value	1.00E-02	1.29E-01	1.24E-03	Skin	
GSE32924	Healthy	8	Log2 Fold change	−1.44	1.78	1.86	Skin	Suárez-Fariñas *et al*.^36^
	AD	13	p-value	0.00E + 00	2.44E-02	3.25E-04	Skin	
GSE36842	Healthy	15	Log2 Fold change	−1.29	1.40	0.16	Skin	Gittler *et al*.^39^
	AD	8	p-value	0.00E + 00	7.20E-13	5.76E-01	Skin*	
GSE5667	Healthy	5	Log2 Fold change	−0.54	0.64	0.39	Skin	Plager *et al*.^40^
	AD	6	p-value	1.00E-01	8.97E-03	2.54E-02	Skin	
GSE6012	Healthy	10	Log2 Fold change	0.37	1.38	0.93	Skin	Mobini *et al*.^41^
	AD	10	p-value	2.60E-01	1.05E-02	4.79E-02	Skin”	
GSE75890	Healthy	8	Log2 Fold change	0.56	0.66	−0.02	Skin	Martel *et al*.^42^
	AD	9	p-value	0.00E + 00	2.75E-01	9.36E-01	Skin^	

*acute skin lesions, “skin lesions from atopic eczema, ^intrinsic skin lesions. Age and gender were not provided or accessible for individual subjects in the gene array cohorts.

### Decreased BLMH results in elevated CXCL8 and GROα release by HaCat cells

**Figure 2:**
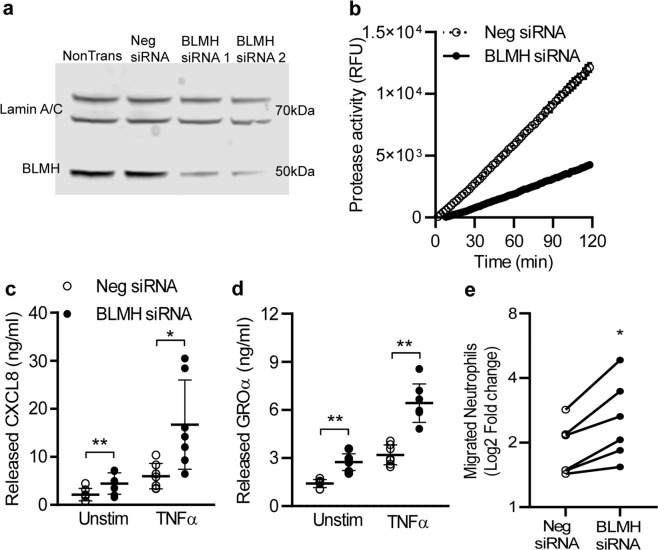
High levels of CXCL8 and GROα is released from BLMH low expressing keratinocytes, with subsequent increased chemotaxis by neutrophils. (**a**) Representative image showing transfection of HaCaT cells with BLMH specific or scrambled negative siRNAs for 24 hours and Western blots analysis of the protease expression (n = 8). Full-length blots are presented in Supplementary Fig. 3a. (**b**) In paired experiments, the protease activity in cell lysates from BLMH knock-down cells and negative control were measured for 2 hours using citrulline-AMC substrate (n = 7). (**c,d**) Supernatants were collected from cells after 24 hours stimulation with or without TNFα and the levels of CXCL8 and GROα were analysed with separate ELISAs (n = 7, one-way ANOVAs with Sidak’s multiple comparisons test). (**e**) The supernatants from negative siRNA or BLMH knock-down cells were used as a chemoattractant in a chemotaxis transwell assay using freshly isolated neutrophils. After 2 hours incubation, the migrated cells in the lower well were collected and stained for 7AAD to enable counting of live cells using an Accuri flow cytometer (n = 6, paired two-tailed Student’s t-tests). The fold change of migrated live neutrophils was determined by normalizing against a negative control (migration towards culture medium). All values represent individual experiments with mean ± standard deviation. Significant P-values are presented as *p < 0.05; **p < 0.01; ***p < 0.001 and ****p < 0.0001.

To investigate the effect of reduced BLMH levels in keratinocytes, we generated BLMH knock-down HaCaT cells by siRNA transfections and confirmed a lowered intracellular protein expression with Western blot and Immunofluorescent staining (Fig. 2A and Supplemental Fig. 1B). Two different sets of BLMH specific siRNAs were used to test knock-down specificity and the more stable siRNA s2002 was subsequently used throughout the experimental procedures. The cellular viability remained at >96% and was not affected by the siRNA transfection or the later TNFα stimulation (Supplemental Fig. 1A). The BLMH knock-down also resulted in a significant reduction of aminopeptidase activity against citrulline-AMC fluorescent substrate (Fig. 2B and Supplemental Fig. 1C), reportedly the most effective substrate for analysing the function of BLMH^6^.

The broad spectrum of different mediators produced and released from HaCaT cells prompted us to perform a screening array where we assessed 36 different cytokines, chemokines and acute phase proteins in supernatants from BLMH knock-down cells compared to control. High levels of serine protease inhibitor Serpin E1 and inflammatory cytokine MIF was detected in cell-free media from both conditions, while supernatants isolated from BLMH knock-down cells contained more CXCL8 and GROα compared to negative control (Supplemental Fig. 1E,F). These results were confirmed in repeated experiments analysing actual levels of CXCL8 and GROα using separate ELISAs (Fig. 2C,D) and also in a separate epithelial cell line BEAS-2b (Supplemental Fig. 1G). In both epithelial cell lines, the increase of CXCL8 and GROα is evident in unstimulated cells and after stimulation with low levels of TNFα, a proinflammatory cytokine present in elevated levels in AD patients^19,20^. CXCL8 and GROα belong to the same family of CXC chemokines and are potent neutrophil chemoattractants^21^. Consequently, significantly more primary neutrophils migrated towards the supernatant from BLMH knock-down cells compared to conditioned media from control cells (Fig. 2E). These results show that a modest increase in the levels of CXCL8 and GROα have functional impact on the migration of neutrophils. Overexpression of BLMH (Supplemental Fig. 2A,B) correlated with a clear increase in protease activity against citrulline (Supplemental Fig. 2C,D). Supernatants collected from these cells contained significantly lower levels of CXCL8 and GROα after stimulation with TNFα (Supplemental Fig. 2E,F), further strengthening the importance of keeping an intact BLMH function to avoid excess release of inflammatory chemokines.

### BLMH low expressing keratinocytes show impaired wound healing capacity in the presence of TNFα

**Figure 3:**
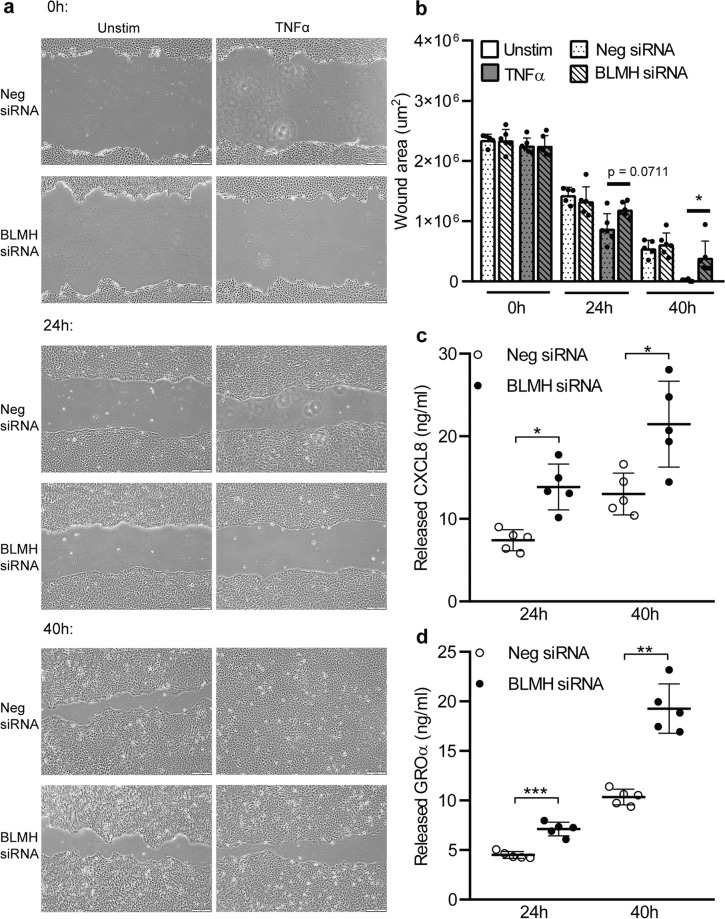
Knock-down of BLMH results in delayed wound healing. (**a,b**) Scratch wound assays were performed on monolayers of siRNA-transfected HaCaT cells and the wound area was analysed after 24 and 40 hours in presence or absence of additional TNFα (5 ng/ml) (n = 6, paired two-tailed Student’s t-tests). Scale bar shows 200 µm. (**c,d**) Supernatants were collected from the scratch wounds after 40 hours and the levels of CXCL8 and GROα were determined using ELISAs (n = 5, one-way ANOVAs with Sidak’s multiple comparisons test). All values represent individual experiments with mean ± standard deviation. Significant P-values are presented as *p < 0.05; **p < 0.01; ***p < 0.001 and ****p < 0.0001.

BLMH is present in the stratum corneum of human skin^16^ and CXCL8 in the stratum corneum serves as an indicator of the severity of inflammation in AD patients^22^. Furthermore, CXCL8 and GROα are the two major chemokines involved in the inflammatory phase of wound healing^23^. Defective wound healing is a common trait in chronic inflammatory skin diseases, elevating the risk for subsequent bacterial infections and allergen penetration^24,25^. To investigate if low levels of BLMH could affect the wound healing capacity of keratinocytes, we performed scratch wound assays on BLMH knock-down HaCaT cells and analysed the wound closure. After 40 hours, HaCaT cells transfected with negative scrambled siRNA effectively closed the wound in the presence of TNFα (Fig. 3A,B). Conversely, BLMH knock-down cells showed a delay in their ability to heal the scratches in presence of inflammatory TNFα. The downregulation of BLMH was maintained throughout the whole scratch wound experiment (unpublished observation) and the levels of CXCL8 and GROα increased over time in the wound supernatants (Fig. 3C,D), indicating a continuous production from the keratinocytes.

### The delayed wound healing is caused by soluble mediators and signals via the CXCR2 receptor

**Figure 4:**
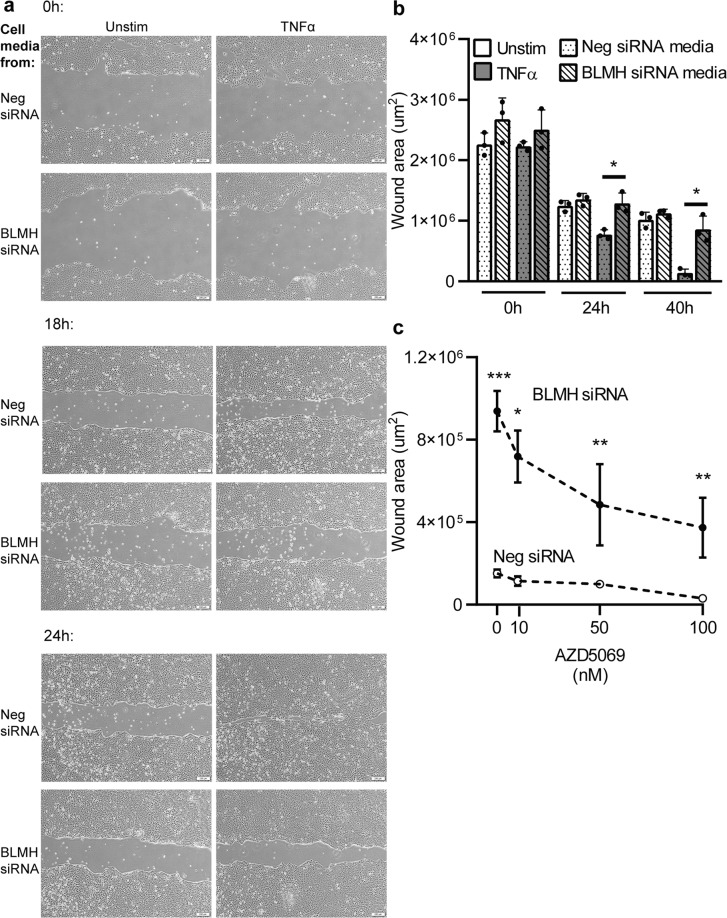
The impaired wound healing is caused by soluble mediators in cell supernatants from BLMH knock-down cells. (**a,b**) Untreated HaCaT cells were cultured into monolayers to approximately 80% confluency. A scratch wound was performed, and supernatants collected from BLMH knock-down cells were added, diluted 1:1 in fresh culture media. The wound areas were analysed after 18 and 24 hours of incubation, showed as representative images (**a**) and as wound area measurements (**b**)(n = 3, paired two-tailed Student’s t-tests). In graph **c**, a specific CXCR2 receptor antagonist was added together with the supernatants at different concentrations and after 30 hours the wound area was determined (n = 4, paired two-tailed Student’s t-tests). All values represent individual experiments with mean ± standard deviation. Significant P-values are presented as *p < 0.05; **p < 0.01; ***p < 0.001 and ****p < 0.0001.

To investigate whether the delayed wound healing was due to intracellular defects caused by the BLMH knock-down or extracellular mediators released from the cells, we cultured wild type HaCaT cells and performed the scratch wound assay in the presence of supernatants collected from BLMH knock-down cells or controls. In supernatants containing low-levels TNFα, the wound afflicted upon the cells with control supernatants was fully healed after 24 hours (Fig. 4A,B), while media from cells expressing low BLMH significantly disrupted the wound healing process. This indicates that the inflammatory mediators released from cells expressing low BLMH regulates the wound repair executed by keratinocytes.

CXCL8 and GROα both bind to the G protein-coupled receptor CXCR2, commonly expressed on epithelial cells and leukocytes. To investigate whether CXCR2 is involved in mediating the delayed wound healing, we treated the HaCaT cells with a selective CXCR2 antagonist (AZD5069) and evaluated the effect on wound healing in the presence of supernatants from BLMH knock-down or control cells. Our results show that blocking the CXCR2 receptor could partly restore the delayed wound healing in the wells containing media from cells expressing low BLMH in a dose dependent manner after 30 hours (Fig. 4C). Together, these findings indicate that the excess release of CXCL8 and GROα by cells expressing low BLMH interferes with the normal wound healing process via CXCR2 signalling.

## Discussion

Dysregulation of proteolytic pathways is now recognized as an underlying cause of several skin pathologies in humans, such as AD, psoriasis and rosacea^2,5^. This holds true for BLMH, a bleomycin-detoxifying protease whose physiological function and substrates remain largely unknown, although its expression and function are known to be suppressed in skin from AD patients. We demonstrate that this reduced expression of BLMH inversely correlates with expression of the chemokines CXCL8 and GROα in lesions from AD patients in a majority of the clinical studies analysed, a difference not seen in healthy individuals. To mimic the AD disease phenotype, we generated a BLMH knock-down keratinocytic cell line (HaCaT) and detected increased release of CXCL8 and GROα from these cells. The mechanism by which BLMH regulates the secretion of these chemokines is unclear but decrease of BLMH did not affect the RNA levels of CXCL8 (Supplemental Fig. 2G,H), indicating that BLMH does not regulate the mechanisms involved in transcription of these chemokines. Kamata *et al*. reported citrulline-containing proteins to be the BLMH substrate of choice^6^, and showed that BLMH is crucial for the degradation of citrullinated filaggrin into natural moisturizing factors. Despite this link to citrullination, blocking the PAD-dependent citrullination in BLMH low expressing keratinocytes using the pan-PAD inhibitor BB-Cl-amide did not affect the secretion of CXCL8 nor GROα (unpublished observation). Even though this observation needs further exploration, it questions the role of citrullination in the regulation of chemokine release by BLMH. Instead, several studies demonstrate that BLMH is localized to the secretory pathways of vesicles, endosomes and ribosomal proteins^13,26,27^. Since chemokines, such as CXCL8 and GROα, are pre-stored and quickly released from secretory vesicles^28–30^, a potential hypothesis is that BLMH interferes with this transportation to disrupt a timely secretion of these key signalling molecules.

Another feature of AD is the impaired wound healing seen as a result of the chronic inflammatory environment. A complex signalling network involving growth factors, cytokines and chemokines plays a role in restoring the skin barrier^31^, where CXCL8 and GROα are maximally expressed at day 1 in the wound bed and correlate with neutrophil infiltration and keratinocyte migration, before subsiding during wound closure^32^. Both chemokines are highly expressed along the bare wound surface where keratinocytes migrate to close the wound, sites that are severely prone to infections by pathogens^33^. We demonstrate that cells expressing low BLMH have a delayed wound healing capacity in the presence of inflammatory TNFα, through soluble factors excreted from the cells. A few broad-spectrum chemokine inhibitors have been tested in animal models including NR58-3.14, which inhibit both CC and CXC chemokines and show potential in improving wound healing^34^ and decreasing leukocyte recruitment *in vivo*^35^. In line with these studies, we show that blocking CXCR2 receptor effectively improves the wound healing process of keratinocytes despite the high levels of CXCL8 and GROα in supernatants collected from BLMH knock-down cells stimulated with low-dose TNFα.

In conclusion, our study presents a novel function of BLMH in contributing to the release of inflammatory chemokines, which results in delayed wound healing by keratinocytes. These findings highlight the importance of maintaining an intact BLMH expression in skin to minimize the inflammatory environment seen in chronic inflammatory skin diseases.

## Methods

### Human blood samples

Blood samples were collected from healthy volunteers. Informed consent was obtained from all blood donors and ethical permit was obtained from Gothenburg ethical review board. All methods were performed in accordance with the Declaration of Helsinki.

### HaCaT cell culture

HaCaT cells, a spontaneously immortalized human keratinocyte line, were cultured in 5% CO_2_ at 37 °C in DMEM (Dulbecco’s Modified Eagle Medium) + 4.5 g/L Glucose + Sodium Pyruvate + Glutamax (Gibco) supplemented with 10% fetal bovine serum (Gibco) + 1% PEST (Gibco). Upon reaching 70–80% confluency, the cells were washed with warm PBS, detached with Accutase (Invitrogen) and seeded into T75 culture flasks or culture plates. For all experiments, 2,5 × 10^4^ and 5 × 10^4^ cells were seeded into 24 and 12 well culture plates, respectively. The culture media was replaced every second day.

### siRNA transfection

To generate a BLMH knockdown in HaCaT cells, small interfering RNAs (siRNAs) from Silencer Select (Invitrogen) was purchased (BLMH specific siRNAs assay ID s2001 and s2002, 4392420, and Silencer Select Negative Control 4390843). siRNAs (10 nM) were mixed with 1 ul Lipofectamine RNAiMAX (Invitrogen) and 98 ul Opti-MEM Reduced Serum Medium (Gibco) and incubated for 30 minutes at room temperature. The siRNA-lipofectamine mix was added dropwise to HaCaT cells plated in 12 well culture plates and incubated overnight. After transfection, the cells were washed with fresh culture media and rested for 4 hours pending further stimulation. A viability check was performed 24 hours after the siRNA transfection using Trypan blue staining in a Cedex HiRes (Ninolab) cell counter.

### Plasmids, DNA preparation and Neon Transfection

An IRES-containing bicistronic vector for expressing the BLMH gene was purchased from Epoch Life Science, Inc., together with the enhanced green fluorescent protein (EGFP) under control of the CMV promoter. The plasmids were amplified in One shot Top10 *E.coli* (ThermoFisher), isolated and purified using Endo-Maxi Free Kit from QIAGEN. DNA purity and concentration were determined spectroscopically with NanoDrop. 1 ug of each vector was digested with restriction enzymes EcoRI and/or NheI and run on an E-gel containing Ethyliumbromide (ThermoFisher) to confirm correct size of the DNA fragments. Cells were electroporated using the Neon Transfection system (Invitrogen). Cultured HaCaT cells were detached using Accutase, counted and washed twice with warm PBS. After a final wash, the cells were resuspended in 30 ul of Resuspension Buffer R (Neon 10 ul Kit, Invitrogen) and mixed with 500 ng of vector diluted in Buffer R. Electroporation was carried out at pulse voltage 1,600, pulse width 10 and pulse number of 3, and the cells were seeded in a 24-well plate with pre-warmed culture media. The EGFP fluorescence was monitored for 72 hours using an Incucyte (Essen BioScience).

### Protein extraction and analysis of protein content

For total protein extraction, HaCaT cells were washed with PBS and lysed in RIPA lysis buffer (ThermoFisher Scientific) supplemented with PhosSTOP (Roche) and cOmplete Protease inhibitor Cocktail (Roche), on ice for 15 minutes. Samples were collected, centrifuged at 14,000 rpm for 10 minutes and supernatants were aliquoted and kept frozen in −80 °C until use. The protein content was determined using Pierce BCA Protein Assay kit (ThermoFisher), according to manufacturer’s protocol.

### Western blot

For Western blot analysis of protein expression, 30 ug of total protein lysates was mixed with NuPAGE LDS Sample buffer (Invitrogen) and NuPAGE Sample Reducing Agent (Invitrogen) and heated for 10 minutes, 70 °C. The samples were loaded onto NuPAGE 4–12% Bis-Tris Protein Gels (Invitrogen) and run with NuPAGE MOPS SDS Running buffer (Invitrogen) according to the NuPAGE Novex electrophoresis program. The proteins were transferred to Nitrocellulose Blotting Membranes (Invitrogen) using NuPAGE Transfer buffer (Invitrogen) containing 20% methanol, followed by blocking with 5% milk in PBS + Tween for 1 hour on shaker. For detection of BLMH, the membranes were incubated cold overnight with Human BLMH Antibody (R&D Systems) 1:1000 dilution in blocking buffer. Next day, the membranes were washed in PBS + Tween for 3 × 5 minutes and stained with Lamin A/C Antibody (Cell Signaling Technology) 1:1,000 dilution in blocking buffer for 1 hour at room temperature. After washing, the membranes were incubated with IRDye Goat anti-Mouse and Donkey anti-Rabbit secondary antibodies (1:10,000 dilution, LI-COR Biosciences) for 1 hour at room temperature. The Western blot was analysed using an Odyssey CLx scanner and the ImageStudio software (LI-COR Biosciences).

### Protease activity assay

To measure the protease activity in HaCaT cells, 30 ug of total protein lysates were transferred into wells of a black 96 well half-area plate (Corning, CLS3694) and 0.1 mM H-citrulline-AMC fluorescent substrate (Bachem, 4019017) was added. For a total volume of 100 ul, assay buffer (50 mM HEPES, 5 mM EDTA, 10 mM DTT dissolved in distilled water) was added to the wells and the fluorescence intensity was read at excitation and emission wavelengths of 380 nm and 460 nm, respectively, using a PHERAstar Plus plate reader (BMG Labtech). The background fluorescence of the citrulline-substrate was subtracted from the lysate-containing wells.

### Detection of soluble inflammatory mediators

Human Cytokine Array Kit (R&D Systems) was used to measure relative levels of inflammatory mediators in cell-free supernatants from HaCaT cells, according to the manufacturer’s protocol. The release of IL-8/CXCL8 and CXCL1/GROα from HaCaT cells was quantified using Human IL-8/CXCL8 DuoSet ELISA and Human CXCL1/GRO alpha DuoSet ELISA (R&D Systems) following the manufacturer’s protocol.

### Neutrophil chemotaxis assay

Blood was obtained from healthy donors and mixed 1:1 with 2% Dextran. After sedimentation of erythrocytes, the leukocytes were separated by density gradient centrifugation. The granulocyte pellet was cleared from remaining erythrocytes by lysing in distilled water and resuspended in RPMI 1640 media supplemented with 5% FCS and 1% PEST. The neutrophil purity was >95% after isolation and determined morphologically with BD Accuri C6 flow cytometer. 1,5 × 10^5^ neutrophils were added to the upper insert of a 12 well transwell plate with 5 µm pore size (Corning). The inserts were placed in the lower well containing 800ul of cell supernatants from siRNA transfected HaCaT cells, diluted 1:8 in RPMI 1640 + 5% FCS + 1% PEST media. After 2 hours of incubation, the upper inserts were removed and 800 ul of the lower media was transferred to FACS tubes. 20 ul of 7AAD (BD Biosciences) was added and incubated cold for 10 minutes. Without washing, 100 ul of the cell media was collected and the 7AAD-negative Neutrophils were counted using BD Accuri C6 flow cytometer.

### Scratch wound assay

HaCaT cells were seeded into 12-well culture plates and incubated until 80% confluency was achieved. Wounds were then inflicted by dragging a sterile 1 ml pipette tip across the monolayer and rinsing off released cells and debris with warm PBS. Fresh culture medium was added with or without conditioned media or recombinant TNFα (5 ng/ml, Peprotech) and images were taken of the wound using Olympus CX31 microscope. Time-lapse images were taken over a 40-hour period and the wound closure was determined by measuring the wound area using Adobe Photoshop.

### RNA isolation and quantitative RT-PCR

Transcription levels of BLMH and CXCL8 were analysed by quantitative RT-PCR. Total RNA from siRNA transfected HaCaT cells was extracted using RNeasy Plus Mini kit (Qiagen), according to the manufacturer’s instructions and the content was determined with Nanodrop. 300 ng of RNA was reverse-transcribed using the High capacity cDNA reverse transcriptase kit (Applied Biosystems). Quantitative RT-PCR was performed with QuantStudio 7 Flex real-time PCR system (Applied Biosystems) using the following TaqMan gene expression assays: BLMH Hs0016671_m1, CXCL8 Hs00174103_m1, EDF1 Hs00610152_m1, GAPDH Hs02786624_g1 and ACTB Hs01060665. The reactions were run in triplicates and Cycle threshold (Ct values) were normalized to housekeeping gene EDF1. Relative quantity calculations were performed using the 2^-ddCt^ method.

### Immunofluorescence

2 × 10^4^ HaCaT cells were seeded onto a 4 well Nunc Lab-Tek Chamber Slide (ThermoFisher) pre-coated with Poly-L-lysine. Next day, the cells were washed with warm PBS twice and fixed with 2% PFA for 5 minutes. After extensive wash with PBS, the cells were blocked for 15 minutes in IHC/ICC Blocking buffer (Low Protein, Ebioscience). The blocking buffer was removed and replaced with primary BLMH antibody (R&D Systems) diluted 1:500 in PBS + 0.1% Triton X + 1% Blocking buffer overnight at 4 degrees. Next day, the slides were washed with PBS and stained with goat anti-mouse IgG, IgM (H + L) Alexa Fluor 488 secondary antibody (ThermoFisher) diluted 1:1,000 in PBS + 0.1% Triton X + 1% Blocking buffer for 1 hour at room temperature. After two more washes with PBS, Hoechst 33342 (Invitrogen) was added diluted 1:10,000 in PBS for 10 minutes at room temperature. The Chamber Slide walls were then removed, and the slide mounted with ProLong Diamond Antifade Mountant (ThermoFisher) onto a cover slip. Images were acquired using an LSM 880 system (Carl Zeiss Microscopy, Germany) with a Zeiss Image Z.1 microscope, Plan-Apochromat 40 x/1,3 objective (Carl Zeiss Microscopy, Germany). Brightness and contrast were adjusted using the Zen software (Black ed. v. 2,3, Carl Zeiss Microscopy, Germany).

### Bioinformatics and statistics

Data are presented as individual experiments with mean ± standard deviation. A meta-analysis of 7 AD cohorts was conducted using OmicsSoft DiseaseLand genomic database (Qiagen). Detailed genomics analysis was performed on data from genomic Project GSE32924 using the probes 202179 (BLMH), 202859 (CXCL8) and 204470 (GROα)^36^. For statistical analyses of multiple comparison tests, one-way ANOVA with Sidak’s multiple comparisons tests were used and for single comparison tests parametric Student’s t-tests were performed. Significant P-values are presented as *p < 0.05; **p < 0.01; ***p < 0.001 and ****p < 0.0001.

## Supplementary information

Supplementary Information
