# Supplementary material for: Bleomycin hydrolase regulates the release of chemokines important for inflammation and wound healing by keratinocytes
Source: Sci Rep. 2019 Dec 31;9:20407. doi: 10.1038/s41598-019-56667-6 (PMC6938525; doi:10.1038/s41598-019-56667-6)
Supplement: Supplementary file 1 — Supplementary Information [file 41598_2019_56667_MOESM1_ESM.pdf]

Bleomycin hydrolase regulates the release of chemokines important for inflammation and wound healing by keratinocytes

Rebecca Riise[1], Lina Odqvist[1], Johan Mattsson[2], Susan Monkley[2], Suado M Abdillahi[1], Christian Tyrchan[3], Daniel Muthas[2], Linda Fahlén Yrlid[1*]

Supplemental figure 1

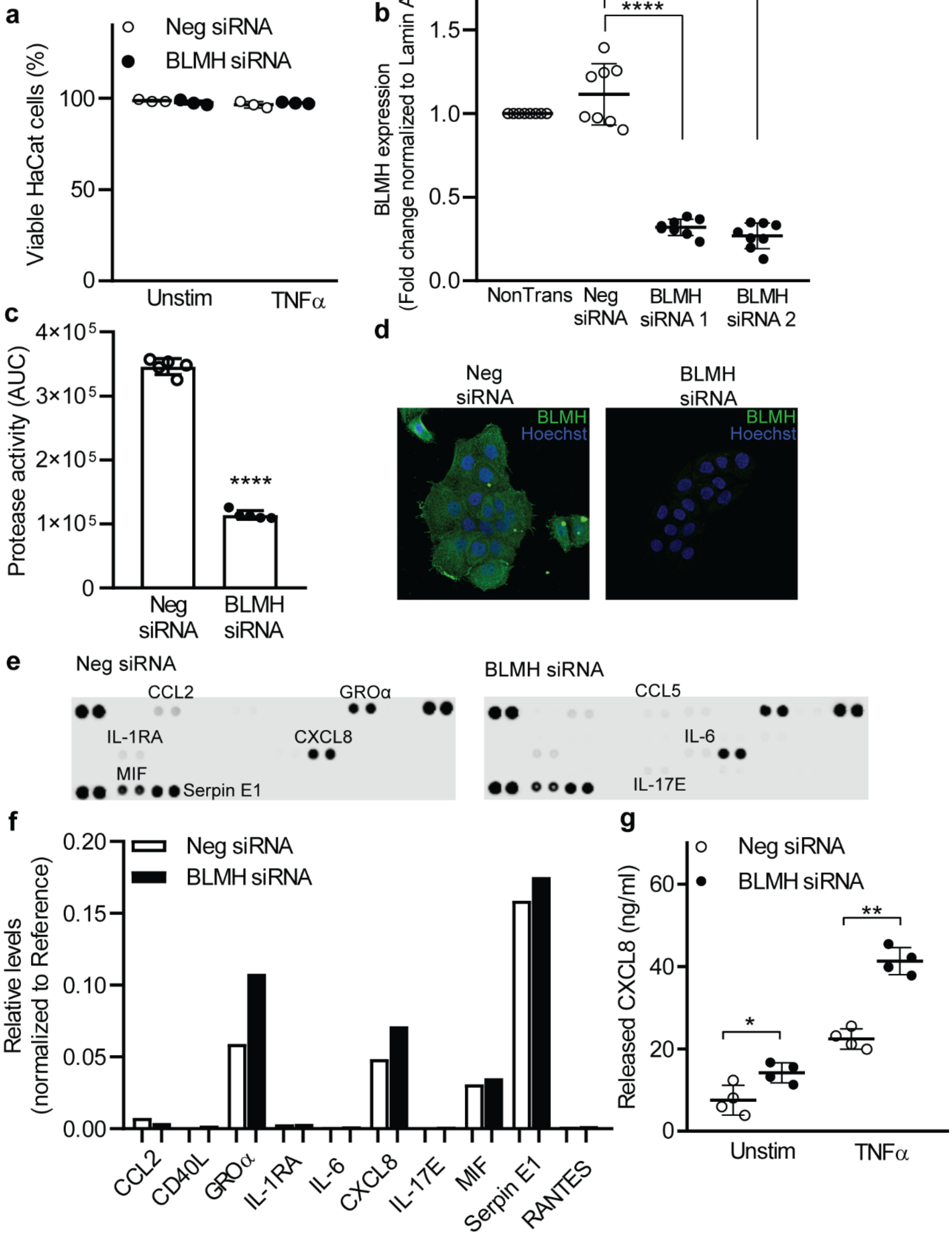

Supplemental figure 1. (**a**) The viability of siRNA transfected cells was analyzed with Trypan blue after stimulation with or without TNFα for 24 hours (n = 3). (**b**) The BLMH protein expression was determined with Western blot after siRNA knock-down using two different siRNA sequences for 24 hours (n = 7, one-way ANOVAs with Sidak's multiple comparisons test). The protein band intensity was normalized to Lamin A/C expression within the same

sample. (**c**) The protease activity in lysates from BLMH low expressing cells versus control was measured against a citrulline-containing substrate and presented as area under the curve (n = 7, two-tailed Student's t-tests). (**d**) Confocal images showing intracellular staining of BLMH and Hoechst after siRNA transfection of HaCaT cells. (**e,f**) Conditioned media from siRNA transfected HaCaT cells were collected after 24 hours and screened for released mediators using Human Cytokine Arrays. (**g**) The lung epithelial cell line BEAS-2b was transfected with BLMH or negative siRNA for 24 hours and stimulated with or without TNFα overnight. Supernatants were collected and the levels of CXCL8 was analyzed with ELISAs (n = 4, one-way ANOVAs with Sidak's multiple comparisons test). All values represent individual experiments with mean ± standard deviation. Significant P-values are presented as $*p < 0.05$; $**p < 0.01$; $***p < 0.001$ and $****p < 0.0001$.

Supplemental figure 2

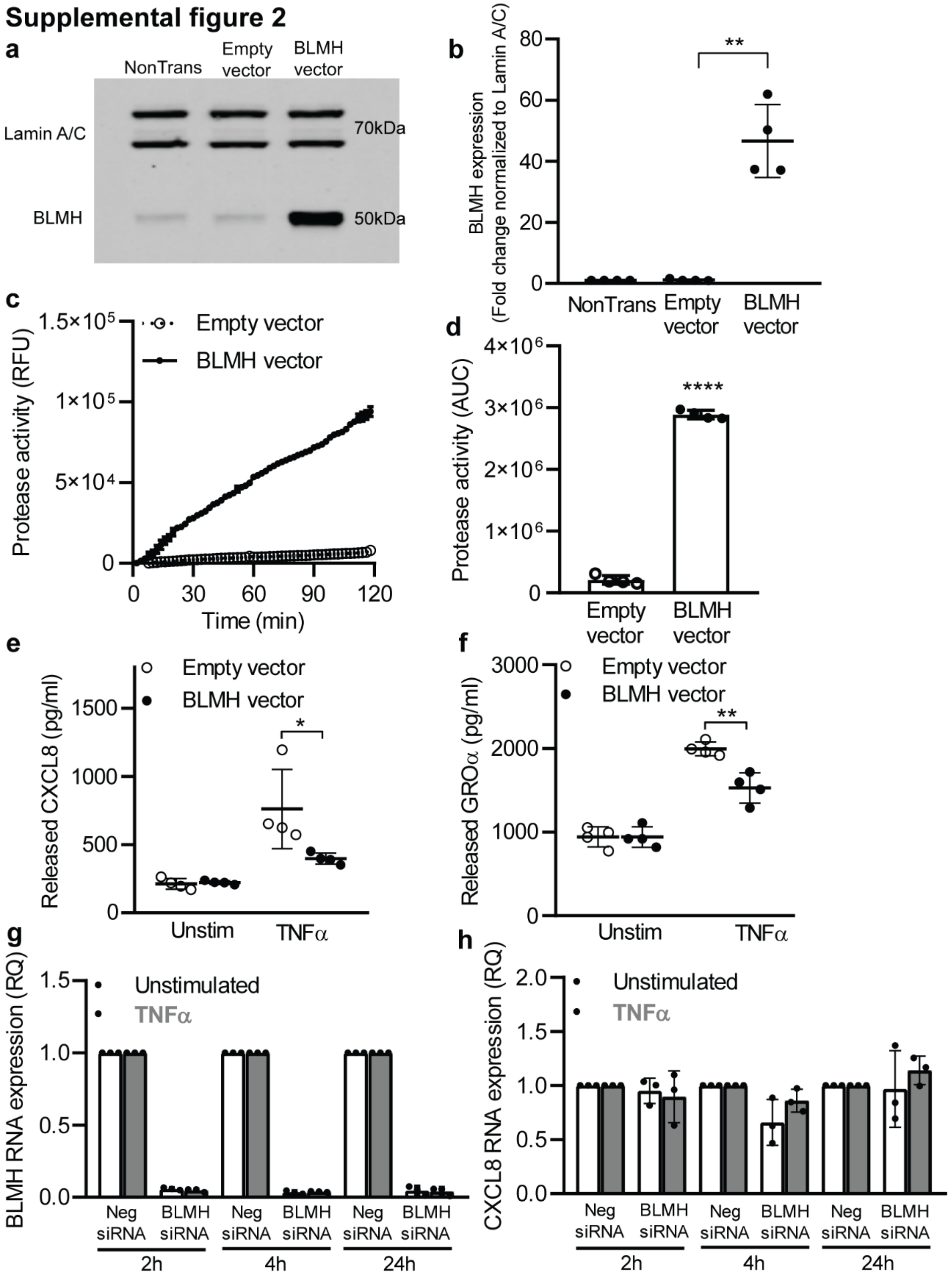

Supplemental figure 2. (**a,b**) HaCaT cells were transfected with an empty or BLMH specific vector for 72 hours and intracellular BLMH levels were determined with Western blot (n = 4, paired two-tailed Student's t-tests). Full-length blots are presented in Supplementary Figure 3b. (**c,d**) The protease activity in these lysates were measured using citrulline-containing substrate for 2 hours (**c**) and presented as area under curve (AUC) (**d**) (n = 4, two-tailed paired Student's

t-tests). (**e,f**) The supernatants were collected and the levels of CXCL8 and GROα were analyzed using ELISAs (n = 4, one-tailed paired Student's t-tests). (**g,h**) HaCaT cells were transfected with siRNAs for 24 hours and stimulated with or without TNFα overnight. RNA was isolated and the gene expression of BLMH and CXCL8 was determined using qPCR (n = 3). All values represent individual experiments with mean ± standard deviation. Significant P-values are presented as $^*p < 0.05$; $^{**}p < 0.01$; $^{***}p < 0.001$ and $^{****}p < 0.0001$.

Supplemental figure 3

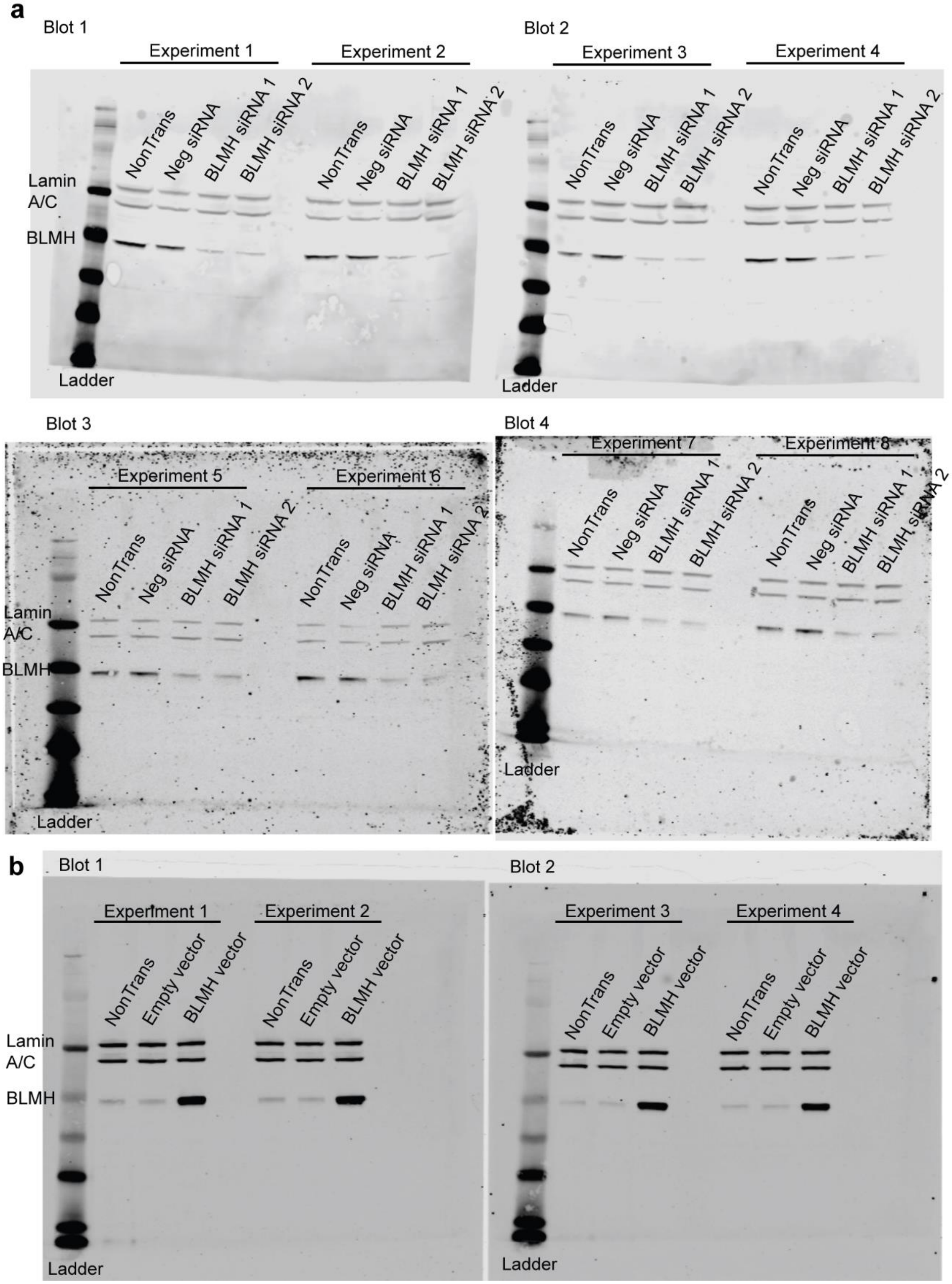

Supplemental figure 3. (**a,b**) Uncropped full-length Western blots showing BLMH and Lamin A/C protein expression after siRNA transfection experiments (n = 8) or BLMH overexpression

assays (n = 4) . Novex Sharp Pre-stained Protein Standard was used as a molecular size marker (ladder).
